# Nursing Institutions, Nurses, and the Women's Jubilee Offering

**Published:** 1887-09-17

**Authors:** Henry C. Burdett


### Nursing Institutions, Nurses, and the Women's Jubilee Offering.

By Henry C. Burdett.

No one can doubt the wisdom and appropriateness of the Queen's decision to devote the money presented to Her Majesty by the women of the British Empire as a jubilee offering to the benefit of nurses and nursing institutions. The wisdom of this gracious decision is borne out by the knowledge that for many months past the nurses of England have been organising themselves through their paper, The Hospital, in the hope of founding a "National Pension Fund for Hospital Officials and Nurses," as a provision against sickness and advancing years. Its appropriateness, by providing for the interests and welfare of the most heroic class of women workers, who devote the best years of their life to the care and nurture of the sick, must be admitted by all. In these circumstances a statement of the present position of nursing and of the needs of nurses and nursing agencies may prove of interest and use in many ways. Before going into details, I desire however, to express a hope that the suggestion of *The Times* will be adopted, and that Her Majesty will be graciously pleased to accept some personal memento of this great outpouring of grateful hearts towards their Queen-Mother on the part of Englishwomen all the world over.

Taking nursing institutions first, it appears that there are some 150 of such establishments, employing probably from 5,000 to 6,000 nurses. Such institutions provide for the supply of trained nurses at fixed rates of payment, varying from one to two guineas per week per nurse, exclusive of travelling expenses, etc. There are, besides, a few district nursing institutions and district nurses, but far too few for the needs of the population. It is, therefore, the fact that, whereas the upper and middle classes can provide themselves at the present day with skilled and certificated nurses in the day of sickness, the lower middle and working classes and the poor are sadly in need of equal facilities under similar circumstances. The requirement of the hour is a greatly increased number of district nurses and nursing institutions in poor town and country places. There is no need for large management expenses or extravagance of any kind, such as one high-sounding institution established years ago under high patronage has introduced. Such extravagances and waste are to be reprobated and stoutly condemned as unnecessary and hurtful in every way. The East London District Nursing Association has set a good example of how to do it, and if similar lines are followed elsewhere, then the great multiplication of district nurses and district nursing institutions under the auspices of the Queen's Jubilee will prove an untold blessing to thousands upon thousands of the sick and poor throughout the country. There has been a feeling expressed from time to time in favour of a new institution, to be called a "Holiday Home for Nurses," where they could go for rest and change when worn out by watching or convalescing from illness. This proposal has been fully discussed by those who have most to do with nurses and their work, and the general and almost unanimous feeling is decidedly against it. Nurses, like other people in such circumstances, require complete rest and change, and such a holiday home would keep their minds constantly upon their work, besides being a centre of gossip for all institutions affiliated with it. Far better adopt the plan of engaging a cottage in the country or near the sea, where some lady or ladies would take an interest in the nurse and provide for her amusement and enjoyment. At least this is the general feeling, and I think it will commend itself to public approval.

So much for nursing institutions, but what of the nurses themselves? Years ago in the columns of *The Times* I commended nursing to gentlewomen as a career which they might usefully and profitably adopt. Miss Sheriff and others took me severely to task for what I then did, but the result has justified my action. From America I hear on the best authority that lady doctors find it so hard to get a living that they are turning to nursing as a profession which will better provide for their comfort and necessities. During the last two months one lady, who has deservedly won for herself a name in the nursing world, has retired upon a pension of several hundreds per annum, while her successor, from a high sense of duty, has nobly decided to put aside her own comfort and predilections by relinquishing her present sphere of usefulness in favour of one where the difficulties and work are serious indeed. Hundreds of gentlewomen enter the nursing profession every year, to the great advantage of the sick, and let us hope of themselves also. I estimate that there are quite 15,000 nurses in this country to-day, of whom from one-half to two-thirds are certificated or hospital-trained nurses Very little has, however, as yet been done for this great army of devoted women. No institutions have any adequate pension and sick-funds, and few have any provision of the kind at all. The general public have no means of deciding for themselves if a nurse is trained or not, and so the incapable and worthless discredit nursing unchecked and to the fullest extent. These two wants—(1) a national pension and sick fund, and (2) a register of trained nurses—have attracted a great deal of attention of late years. About six years ago I issued a circular to all the chief hospitals and nursing institutions asking certain questions, with the view of establishing a pension fund upon a sound financial basis. The figures and facts thus collected were submitted to two eminent actuaries, the late Cornelius Walford and Mr. King, of the Atlas office. They prepared an elaborate scheme providing for a pension and sick fund for nurses and hospital officials, including the acceptance of donations from members of the public as bonuses in favour of the general fund or of any particular nurse. The scheme is based upon provident principles, and contemplates the co-operation of the managers of the various hospitals and nursing institutions and the utilisation of existing organisations, so as to reduce the expenses of management for the fund to a merely nominal sum per annum. Some 2,500 people gave in their conditional promise to join such a fund, but it has not as yet been established, because the *minimum* average annual payment required to secure a pension of £25 per annum at 50 or 55 years of age was £5, a sum equal to 25 per cent. of the nurse's salary. This money nurses could not pay, owing to the claims made upon them by their families and friends and the hospital authorities, who do not in many cases provide washing for the nursing staff. If washing was always provided, or should the committees of the various institutions consent to give annually a sum of from 5 per cent. to 10 per cent. on the present total paid for salaries to the nursing staff as a bonus to the fund, the pension fund would at once be a success. That it would prove an economy to the institutions in the long run, few who are familiar with the facts will doubt; and with the view of testing the feeling of the hospital officials and nurses as to the desirability of establishing such a fund, The Hospitals Association have opened a list for the registry of the names of those who wish to join. During the last few weeks names have been received from 90 institutions, and the columns of THE HOSPITAL afford evidence that names are coming in in increasing numbers every week. Some of the nursing institutions have sent in as many as 70 names, while six hospital secretaries, 53 matrons, lady superintendents, and assistants, and upwards of 500 nurses, have already registered their names as supporters of a provident pension and sick fund. In these circumstances the decision of the Queen to devote the surplus of the Women's Jubilee Offering to nurses and nursing institutions will be welcomed by the hospital authorities, and especially by the nurses and their officers, as an unmixed blessing. There should be no further delay in making the "National Pension Fund for Hospital Officials and Nurses" a financial and popular success. Where those chiefly concerned have displayed so much anxiety and interest in the movement, the general public will not be slow to follow, especially when Her Majesty has blessed the undertaking by selecting it as one of the institutions which she desires to see established in commemoration of her jubilee. Anyone interested will render good service by sending name and address to the secretary of The Hospitals Association, Norfolk House, Norfolk Street, Strand, W.C. Of course the arrangements will be entirely in the hands of any committee the Queen may appoint, and the whole of the papers and information collected will be placed entirely at their disposal. So far as the register for trained nurses is concerned, The Hospitals Association have taken up the movement with spirit, by drawing up rules and opening a register at Norfolk House, which will be under the supervision of the Sectional Committee of Nursing and Domestic Management, of which Mrs. Fenwick (*née* Manson) is the chairman.

In these circumstances, it is to be hoped that nurses will avail themselves of the present great opportunity by registering their names at Norfolk House without delay. The Press can greatly help this movement by making the fact of the opening of the register widely known, and much praise is due to the *British Medical Journal* for its steadfast advocacy of the cause of the nurses. The *Journal* recently expressed the opinion that a provident system for nurses upon a sound actuarial basis would be of the greatest possible advantage to them. Such an institution, if once the advantages it offers could become widely known, might quickly become to a large extent self-supporting; yet there would always be scope for external assistance. I have shown that this view has been put into a practical and working scheme, and I heartily agree with the *Journal* that "probably no more useful destination could be found for a part at least of that jubilee fund, which may be known in future as Queen Victoria's Bounty." The work of many years seems on the eve of successful completion, and I only hope that at length nurses and nursing institutions will receive that substantial recognition and extension which justice and the national interests alike demand.

